# Supplementary material for: Resolving the full spectrum of human genome variation using Linked-Reads
Source: Genome Res. 2019 Apr;29(4):635–45. doi: 10.1101/gr.234443.118 (PMC6442396; doi:10.1101/gr.234443.118)
Supplement: Supplemental Material [file supp_gr.234443.118_Supplemental_longranger-2.2.zip › longranger-2.2.tar.bz2!/lr2.2-public/lib/stan/cmdstan/stan/doc/api/html/col_8hpp_source.html]

Stan: src/stan/math/matrix/col.hpp Source File


|  |  |
| --- | --- |
|  | Stan  2.5.0  probability, sampling & optimization |


- Main Page
- Related Pages
- Namespaces
- Classes
- Files

- File List
- File Members

All Classes Namespaces Files Functions Variables Typedefs Enumerations Enumerator Friends Macros Pages

- src
- stan
- math
- matrix

col.hpp

Go to the documentation of this file.

1 #ifndef STAN\_\_MATH\_\_MATRIX\_\_COL\_HPP

2 #define STAN\_\_MATH\_\_MATRIX\_\_COL\_HPP

3

4 #include <stan/math/matrix/Eigen.hpp>

5 #include <stan/math/error\_handling/matrix/check\_column\_index.hpp>

6

7 namespace stan {

8  namespace math {

9

21  template <typename T>

22  inline

23  Eigen::Matrix<T,Eigen::Dynamic,1>

24  col(const Eigen::Matrix<T,Eigen::Dynamic,Eigen::Dynamic>& m,

25  size\_t j) {

26  stan::math::check\_column\_index("col(%1%)",j,m,"j",(double\*)0);

27  return m.col(j - 1);

28  }

29

30  }

31 }

32 #endif

check\_column\_index.hpp

stan::math::check\_column\_index

bool check\_column\_index(const char \*function, size\_t i, const Eigen::Matrix< T\_y, R, C > &y, const char \*name, T\_result \*result)

Return true if the specified index is a valid column of the matrix.

**Definition:** check\_column\_index.hpp:25

stan::math::col

Eigen::Matrix< T, Eigen::Dynamic, 1 > col(const Eigen::Matrix< T, Eigen::Dynamic, Eigen::Dynamic > &m, size\_t j)

Return the specified column of the specified matrix using start-at-1 indexing.

**Definition:** col.hpp:24

Eigen.hpp


---

[ Stan Home Page ]

*© 2011–2014,
Stan Development Team.*
